# Artemisinin derivatives versus quinine in treating severe malaria in children: a systematic review

**DOI:** 10.1186/1475-2875-7-210

**Published:** 2008-10-17

**Authors:** George PrayGod, Albie de Frey, Michael Eisenhut

**Affiliations:** 1 National Institute for Medical Research, P.O Box 1462, Mwanza, Tanzania; 2 School of Public Health, University of the Witwatersrand, 7 York Road, Parktown 2193, Johannesburg, South Africa; 3 Luton & Dunstable Hospital NHS Foundation Trust, Lewsey Road, Luton, LU4 0DZ, UK

## Abstract

**Background:**

The efficacy of intravenous quinine, which is the mainstay for treating severe malaria in children, is decreasing in South East Asia and Africa. Artemisinin derivatives are a potential alternative to quinine. However, their efficacy compared to quinine in treating severe malaria in children is not clearly understood. The objective of this review was to assess the efficacy of parenteral artemisinin derivatives versus parenteral quinine in treating severe malaria in children.

**Methods:**

All randomized controlled studies comparing parenteral artemisinin derivatives with parenteral quinine in treating severe malaria in children were included in the review. Data bases searched were: The Cochrane Central Register of Controlled Trials (The Cochrane Library Issue 4, 2007), MEDLINE (1966 to February 2008), EMBASE (1980 to February 2008), and LILACS (1982 to February 2008). Dichotomous variables were compared using risk ratios (RR) and the continuous data using weighted mean difference (WMD).

**Results:**

Twelve trials were included (1,524 subjects). There was no difference in mortality between artemisinin derivatives and quinine (RR = 0.90, 95% CI 0.73 to 1.12). The artemisinin derivatives resolved coma faster than quinine (WMD = -4.61, 95% CI: -7.21 to -2.00, fixed effect model), but when trials with adequate concealment only were considered this differences disappeared. There was no statistically significant difference between the two groups in parasite clearance time, fever clearance time, incidence of neurological sequelae and 28^th ^day cure rate. One trial reported significantly more local reactions at the injection site with intramuscular quinine compared to artemether. None of the trials was adequately powered to demonstrate equivalence.

**Conclusion:**

There was no evidence that treatment of children with severe malaria with parenteral artemisinin derivatives was associated with lower mortality or long-term morbidity compared to parenteral quinine. Future studies require adequately powered equivalence trial design to decide whether both drugs are equally effective.

## Background

Malaria remains a major public health problem globally. It is estimated that each year there are about 350–500 million clinical cases of malaria, which result in more than 1 million deaths [1]. About 80% of these deaths occur in sub-Saharan Africa among children aged below five years [1,2]. Therefore, children are the population group with the highest malaria case fatality rate.

Over the years quinine has been the mainstay for treating severe malaria in children, but despite its high efficacy the case fatality rate for severe malaria in children can be as high as 40% [3]. Additionally, in recent years there have been concerns that the efficacy of quinine is declining in some parts of South East Asia and quinine resistance has been documented in Africa [4-7]. This led to the launch of a series of trials to find drugs that are suitable alternatives to quinine. The studies focused on artemisinin derivatives [8-19]; a group of drugs with no known *Plasmodium falciparum *resistance and which act faster than all known malaria drugs [20]. Pharmacokinetic studies in children with severe *P. falciparum *malaria, particularly with metabolic acidosis, however, showed variable and unpredictable absorption of artemether after intramuscular injection a problem not encountered with intramuscular quinine [21,22].

Because the findings from individual studies were inconclusive, two reviews have been conducted to clarify the efficacy of artemisinin derivatives versus existing drugs used for severe malaria [23,24]. The first looked at the efficacy of artemether versus quinine in treating severe malaria [23] and the second evaluated the efficacy of artemisinin derivatives versus standard drugs used for treating severe malaria (e.g. quinine, chloroquine, and others)[24]. The first review showed that there was no significant difference between artemether and quinine in mortality rate (OR = 0.76, 95% CI 0.5 to 1.14, random effects model)[23]. The second review showed that artemisinin derivatives reduced mortality compared to quinine, but the difference was marginal when data from high quality trials only were considered (OR = 0.72, 95% CI 0.54 to 0.96, random effects model) [24]. While these findings seem to suggest that artemisinin derivatives are either equal to, or have marginal advantage over quinine in mortality reduction, both reviews did not consider the efficacy of the drugs on severe malaria in children separately. They pooled data from adults and children. Therefore, it is difficult to apply the findings directly to children; the group, which possibly respond to malaria drugs differently from adults. The objective of this review was to assess the available evidence on the efficacy of parenteral artemisinin derivatives versus quinine in treating severe malaria in children.

## Methods

Studies were considered for inclusion in the review if they were randomized controlled trials comparing one parenteral artemisinin derivative (artemether, β-arteether/artemotil or artesunate) with parenteral quinine in children aged between 0 to 16 years or if trials stated that they included children only. The participants had to have severe malaria as defined by the World Health Organization (WHO) [25]. Trials including both adults and children were excluded. Studies were not considered if: an artemisinin derivative was combined with another antimalarial and compared with quinine, comparison was between two or more artemisinin derivatives or comparison was between regimens of one artemisinin derivative. The review's primary outcome was mortality while parasite clearance time (PCT), fever clearance time (FCT), coma resolution time (CRT), incidence of neurological sequelae, 28^th ^day cure rate and incidence of adverse effects were the secondary outcomes.

Electronic databases and other sources were used to search for studies to include in the review. The electronic databases searched were: The Cochrane Central Register of Controlled Trials (The Cochrane Library Issue 4, 2007), MEDLINE (1966 to February 2008), EMBASE (1980 to February 2008), and LILACS (1982 to February 2008). With the support of a Cochrane information specialist a comprehensive search strategy for each database was developed using both controlled vocabulary terms and free text words to ensure that all potential articles regardless of language or publication status were retrieved. The following key words were used: artemisinins, dihydroartemisinins, artemether, artesunate, β-arteether, artemotil, quinine, malaria, "cerebral, malaria", "severe malaria" complicated malaria, "malaria, falciparum", and randomized controlled trials. Other sources searched included: The proceedings of the 11^th ^annual scientific conference (with seminar on malaria) of the Tanzania National Institute for Medical Research conducted between 4^th ^to 7^th ^February 1993, in Arusha, Tanzania. In addition, 26 malaria researchers across Africa, Asia, Europe and America were contacted to find out if they had any information on published, unpublished or ongoing trials for possible inclusion in the review. The reference lists of the retrieved articles were also searched for more articles.

After the literature search was completed the results were sorted to include abstracts, which had the potential of being included in the review. Complete articles of the potential abstracts were either retrieved or ordered. If a trial was published more than once, only one publication was presented for assessment and if an interim analysis of a particular major trial was published, only the final publication was presented for assessment. GP and AD assessed the articles for their suitability for inclusion in the review.

Then all included studies were assessed by GP and AD for their quality in design and conduct. Generation of allocation sequence, allocation concealment, blinding and loss to follow up, were used to assess the quality of studies as recommended previously [26].

GP extracted data using special forms, and then it was analysed using STATA version 8.2. Binary outcomes were compared by risk ratio (RR) and continuous outcomes by weighted mean difference (WMD). The 95% confidence interval (CI) was used and p value < 0.05 was assumed to be showing a statistically significant difference.

To assess heterogeneity, forest plots were visually examined (for overlapping confidence intervals) and the chi-squared test for heterogeneity with p value < 0.05 was assumed to be showing significant heterogeneity among trials. The fixed effect model was employed in pooling data where there was no evidence of heterogeneity and where there was evidence of heterogeneity, the random effects model was used instead. Because there was significant heterogeneity in parasite clearance time, fever clearance time and 28^th ^day cure rate; it was decided to explore the study setting (Asian versus non-Asian studies) as a possible source of heterogeneity. The rationale for choosing this characteristic was that in Asia, evidence suggested that the level of quinine resistance was higher than on other continents and therefore artemisinin derivatives were likely to be comparably more efficacious in Asia than on other continents; a situation that could have introduced heterogeneity in pooled data. Also sensitivity analysis based on adequacy of concealment was carried out for all outcomes. Data on adverse effects were summarized.

## Results

### Description of studies

Twelve randomized controlled trials involving 1524 children met the inclusion criteria and were included in the review [8-19]. Details of these studies are found in Table 1. They were conducted in nine countries i.e. Nigeria, Kenya, Zambia, Cameroon, Malawi, Gambia, Sudan, India and Vietnam and published between 1993 and 2003. Trials participants were children aged from 0 to 15 years, inclusion criterion in nine trials was cerebral malaria[8-10,12-16,18] and in 3 trials any form of severe malaria [11,17,19]. Nine trials [8-10,12-14,17-19] used intramuscular artemether while one trial used intramuscular artesunate [11]. Two trials used intramuscular artemotil/β-arteether [15,16]. The duration of artemisinin derivatives treatment ranged from 3–6 days while that for quinine ranged from 1 to 7 days. In one trial, quinine was administered intramuscularly [10] and in the rest it was administered intravenously [8,9,11-19].

**Table 1:** Trial participants, interventions and outcomes

**Reference**	**Participants**	**Interventions (drugs, route of administration and treatment duration)**	**Outcomes**
[8]	54 children aged 1 to 5 years in Nigeria	(1) IM Artemether 3.2 mg/kg in the first day, then 1.6 mg/kg for 4 days	Mortality, parasite clearance time, fever clearance time, coma resolution time, 28^th ^day cure rate, adverse effects & neurological sequelae
		(2) IV Quinine 20 mg/kg loading dose then 10 mg 8 hrly for 7 days. This was changed to oral dose when patient was able to drink	

[9]	160 children aged 5 month to 12 years in Kenya	(1) IM Artemether 3.2 mg/kg in the first day, then 1.6 mg/kg for 2 days. One dose of SP was given after parasite clearance.	Mortality, parasite clearance time, fever clearance time, coma resolution time, adverse effects & neurological sequelae
		(2) IV Quinine 20 mg/kg loading dose then 10 mg 8 hrly for at least 3 days. Then one dose of SP was given after parasites clearance and when the patient was able to drink	

[10]	576 children aged 1 to 9 years in Gambia	(1) IM Artemether 3.2 mg/kg in the first day, then 1.6 mg/kg for 3 days	Mortality, parasite clearance time, fever clearance time, coma resolution time, 28^th ^day cure rate, adverse effects & neurological sequelae
		(2) IM Quinine 20 mg/kg loading dose then 10 mg 8 hrly for 5 days. This was changed to oral dose when the patient was able to drink	

[11]	72 children aged 3 months to 14 years in Vietnam	(1) IM Artesunate 3 mg/kg initially and 2 mg/kg at 12, 24,48 and 72 hrs, followed by oral mefloquine 15 mg/kg at 96 hours	Mortality, parasite clearance time, fever clearance time, coma resolution time, duration of hospital stay, adverse effects & neurological sequelae
		(2) IM Quinine 20 mg/kg loading dose then 10 mg 8 hrly for 7 days, followed by single dose of pyrimethamine/sulfadoxine on day 7	

[12]	164 children in Malawi (age range not stated)	(1) IM Artemether 3.2 mg/kg in the first day, then 1.6 mg/kg for 4 days	Mortality, parasite clearance time, fever clearance time, coma resolution time, coma resolution time, 28^th ^day cure rate, adverse effects & neurological sequelae
		(2) IV Quinine(20 mg/kg loading dose then 10 mg 8 hrly for 7 days, changed to oral dose when patient was able to drink	

[13]	37 children in Nigeria (age range not stated)	(1) IM Artemether 3.2 mg/kg starting dose, then 1.6 mg/kg 12 hours later, then 1.6 mg/kg per day for 2 days	Mortality, percentage of children with parasites clearance at day 7, fever clearance time, coma resolution time & neurologic sequelae
		(2) IV Quinine 10 mg/kg initial dose then 10 mg 8 hrly for 7 days, changed to oral dose when patient was able to drink	

[14]	103 children aged 1 to 5 years in Nigeria	(1) IM Artemether 3.2 mg/kg in the first day, then 1.6 mg/kg for 4 days	Mortality, parasite clearance time, fever clearance time, coma resolution time, 28^th ^day cure rate & neurological sequelae
		(2) IV Quinine 20 mg/kg loading dose then 10 mg 8 hrly for 7 days, changed to oral dose when patient was able to drink	

[15]	92 children aged 0–10 years in Zambia	(1) IM β-Arteether 3.2 mg/kg in the first day, then 1.6 mg/kg for next 4 days	Mortality, parasite clearance time, fever clearance time, coma resolution time, adverse effects & neurological sequelae
		(2) IV Quinine (20 mg/kg loading dose then 10 mg 8 hrly for 7 days, changed to oral dose when patient was able to drink	

[16]	102 children aged 0–10 years in Cameroon	(1) IM β-Arteether 3.2 mg/kg in the first day, then 1.6 mg/kg for 4 days	Mortality, parasite clearance time, fever clearance time, coma resolution time, adverse effects, 28^th ^cure rate & neurological sequelae
		(2) IV Quinine 20 mg/kg loading dose then 10 mg 8 hrly for 7 days, changed to oral dose when patient was able to drink	

[17]	41 children in Sudan (age range not stated)	(1) IM Artemether 3.2 mg/kg in the first day, then 1.6 mg/kg for t 4 days	Mortality, parasite clearance time, fever clearance time, coma resolution time & adverse effects
		(2) IV Quinine 20 mg/kg loading dose then 10 mg 8 hrly for 7 days, changed to oral dose when patient was able to drink	

[18]	77 children aged 3 months to 15 years in Sudan	(1) IM Artemether 1.6 mg/kg, repeated after 12 hrs, then daily for 4 days	Mortality, parasite clearance time, fever clearance time, coma resolution time & 28^th ^day cure rate
		(2) IV Quinine 10 mg/kg 8 hrly for 7 days, changed to oral dose when patient was able to drink	

[19]	46 children aged 0–14 years from India	(1) IM Artemether 3.2 mg/kg in the first day, then 1.6 mg/kg for 5 days	Mortality, parasite clearance time, fever clearance time, coma resolution time & adverse effects
		(2) IV Quinine (20 mg/kg loading dose then 10 mg 8 hrly for 7 days, changed to oral dose when patient was able to drink	

### Methodological quality of included trials

Methods used in generation of allocation sequence were adequate in six trials [9,10,12,14-16] and were not clear in the remaining trials [8,11,13,17-19]. Eight trials had adequate allocation concealment [9-12,14-17] and thus they were of high quality. In one trial the methods used to conceal the allocation were inadequate [19] and in the other three the allocation concealment methods used were unclear [8,13,18]. In three trials, the procedures were described as open [8,9,12]. In four trials, it was stated that there was blinding of microscopists [10,15,17,19]. In the remaining trials there was no description with regard to blinding and indeed trial procedures precluded any blinding.

In three trials, there were no reports of loss to follow up or exclusion from analysis for the primary outcome [13,17,19]. In seven other trials, loss to follow-up or exclusion from analysis was below 10% [8,11,10,14-16,18]. A trial in Malawi excluded from analysis 10.3% (19/183) of study participants [12] and another trial in Kenya excluded from analysis 20% (40/200) of participants initially included in the study [9]. And only three trials described how the sample size required to detect statistical significant differences in outcomes among treatment groups was achieved [10,12,15].

### Mortality

**Figure 1:**
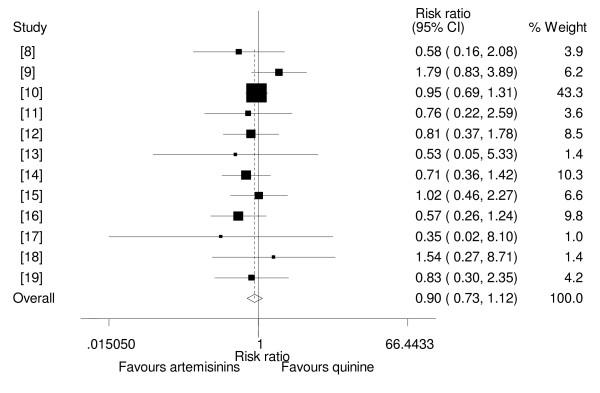
**Meta-analysis of effect of artemisinin derivatives versus quinine on mortality (fixed effect model).** Weight of study was expressed by size of square.

**Figure 2:**
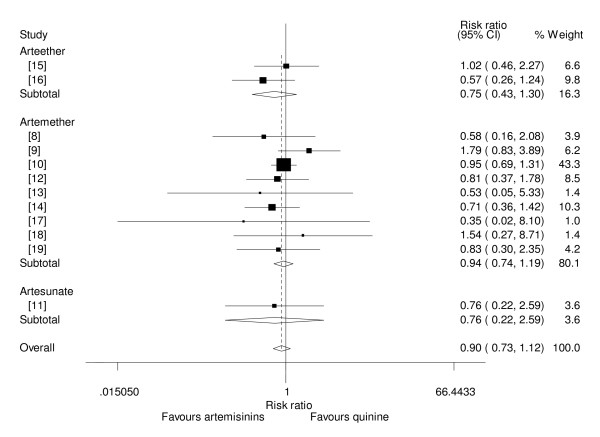
**Subgroup meta-analysis of effect of β-arteether, artemether and artesunate versus quinine on mortality (fixed effect model).** Weight of study was expressed by size of square.

Nine trials [8-12,14-16,19] were designed to measure mortality rate with artemisinin derivatives and quinine as a primary outcome, whilst three trials were not designed to measure mortality as a primary outcome but did report the mortality rates with the two drug groups [13,17,18]. Nine trials showed that artemisinin derivatives had lower mortality than quinine, but none of these results were statistically significant [8,10-14,16,17,19]. Two other trials showed that quinine had a reduced mortality compared to artemisinin derivatives, but the differences were not statistically significant [9,15]. In one trial, a significance test for the difference in mortality rates in the two groups was not stated, but the rates looked comparable (7.8% for artemether and 5% for quinine)[18]. The pooled analysis showed that, compared to quinine, administration of artemisinin derivatives in children with severe malaria was not associated with a reduced mortality(RR = 0.90, 95%CI: 0.73 to 1.12, fixed effect model)(Figure 1). Sub-group analysis showed that, of the three artemisinin drugs studied i.e. β-arteether, artemether and artesunate none was better than quinine in mortality reduction (RR = 0.75, 95%CI: 0.43 to 1.30, fixed effect model), (RR = 0.94, 95%CI: 0.74 to 1.19, fixed effect model) and (RR = 0.76, 95%CI: 0.22 to 2.59, fixed effect model) respectively (Figure 2). Sensitivity analysis based on adequacy of concealment, showed that there was no statistical difference in mortality between artemisinin derivatives and quinine in studies with adequacy of concealment (RR = 0.92, 95%CI: 0.73 to 1.15, fixed effect model).

### Parasite clearance time

Six studies showed no statistically significant difference in mean parasite clearance times between artemisinin derivatives and quinine [8,14-18], whilst one study showed that mean parasite clearance time was shorter in the artemisinin derivative than quinine[19]. Four studies, all with adequate concealment, which reported parasite clearance times as median, found that artemisinin derivatives cleared parasites faster than quinine [9-12]. One trial showed that the percentage of children with parasite clearance at day 7 was significantly higher in the quinine group than in the artemether group [13]. Analysis based on type of drug used showed that of the two drug groups which reported parasite clearance time as mean i.e. β-arteether and artemether none of these cleared parasites faster than quinine.

**Figure 3:**
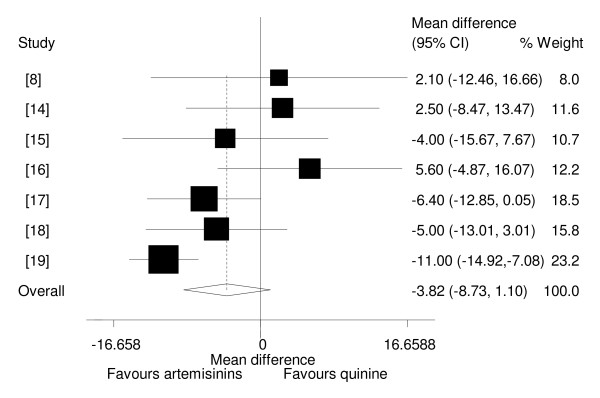
**Meta-analysis of effect of artemisinin derivatives versus quinine on parasites clearance time (random effect model).** Weight of study was expressed by size of square.

Overall, pooled analysis showed that artemisinin derivatives did not clear parasites faster than quinine (WMD = -3.82, 95%CI: -8.73 to 1.10, random effect model). (Figure 3). Also, sensitivity analysis showed that artemisinin derivatives did not clear parasites faster in trials, which were adequately concealed (-1.49, 95% CI -7.36 to 4.39, random effect model). Subgroup analysis revealed heterogeneity between Asian and African studies and showed parasite clearance time was shorter for artemisinin derivatives in Asian studies, but not in African studies ((WMD = -11.0, 95% CI: -14.92 to -7.08, random effect model) and (WMD = -2.46, 95% CI: -6.43 to 1.51, random effect model) respectively).

### Fever clearance time

Of the eight studies which reported fever clearance times, six showed no statistical significant difference in fever clearance times among the two interventions [8,14-16,18,19], while two studies showed that artemisinin derivatives cleared fever faster than quinine[13,17]. Of the four trials which reported fever clearance times as median, three showed no statistical difference in clearance times of the two drug groups [9-11] and one reported that artemether cleared fever faster than quinine [12]. Analysis by the type of artemisinin used showed that neither β-arteether nor artemether cleared fever faster than quinine.

**Figure 4:**
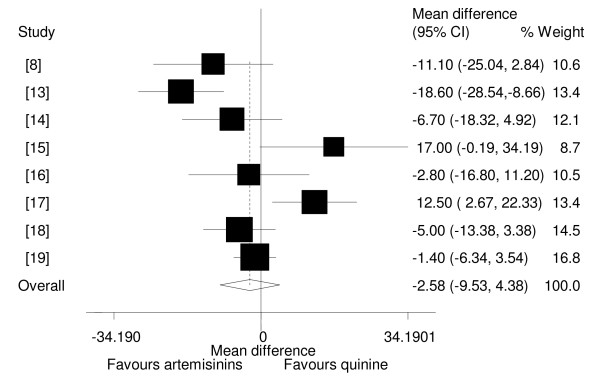
**Meta-analysis of effect of artemisinin derivatives versus quinine on fever clearance time (random effect model).** Weight of study was expressed by size of square.

The pooled analysis showed that artemisinin derivatives did not clear fever faster than quinine (WMD = -2.58, 95%CI: -9.53 to 4.38, random effect model)(Figure 4). Analysis among trials with adequacy of concealment, also showed no statistical significant difference between artemisinin derivatives and quinine (WMD = 4.60,95%CI -6.61 to 15.80, random effect model). Subgroup analysis revealed no evidence of heterogeneity between Asian and African studies ((WMD = -1.40, 95%CI: -6.34 to 3.54, random effect model) and (WMD = -2.64, 95%CI: -11.76 to 6.47, random effect model) respectively).

### Coma resolution time

Among eight studies which reported mean coma resolution times, six trials showed no statistically significant difference between artemisinin derivatives and quinine [8,14-18], whilst the other two trials showed that coma resolution time was significantly shorter in the artemisinin group compared to the quinine group [13,19]. Among four trials which reported median coma resolution times only one trial showed that quinine resolved coma faster than the artemether [10], others showed there were no differences[9,11,12]. Analysis by type of artemisinin drug used showed that artemether, but not β-arteether resolved coma faster than quinine.

**Figure 5:**
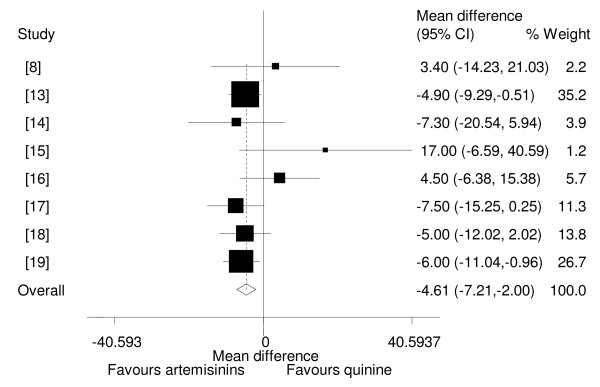
**Meta-analysis of effect of artemisinin derivatives versus quinine on coma resolution time (fixed effect model). **Weight of study was expressed by size of square.

Overall pooled analysis showed that artemisinin derivatives resolved coma faster than quinine (WMD = -4.61, 95% CI: -7.21 to -2.00, fixed effect model (Figure 5). This superiority of artemisinins over quinine was no longer significant when only trials with adequate allocation concealment were considered (-3.00, 95% CI -8.54 to 2.53, fixed effect model).

### Incidence of neurological sequelae

**Figure 6:**
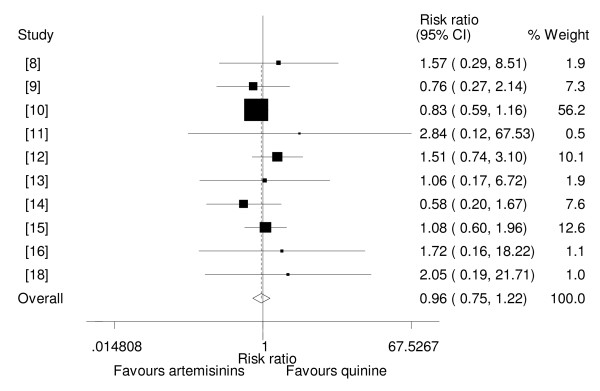
**Meta-analysis of effect of artemisinin derivatives versus quinine on incidence of neurological sequelae (fixed effect model).** Weight of study was expressed by size of square.

Ten trials reported or had data on incidence of neurological sequelae at discharge or at day seven [8-16,18]. The sequelae reported included motor deficits, severe hypotonia, aphasia, abnormality in gait, cortical-deafness, blindness, learning difficulties, hallucinations, hemiplegia and quadriparesis. Two other trials reported that neurological sequelae had not been observed during follow up [17,19]. Of the three artemisinin drugs studied i.e. β-arteether, artemether and artesunate none had less neurological sequelae than quinine. When the data from all ten studies were pooled together the results showed that there was no statistical difference among the two groups for this outcome (RR = 0.96, 95%CI: 0.75 to 1.22, fixed effect model)(Figure 6). When adequately concealed trials only were considered the findings were similar (RR = 0.93, 95%CI: 0.72 to 1.20, fixed effect model)

### 28^th ^day cure rate

Six trials reported or had data that could be extracted on 28^th ^day cure rates [8,10,12,14,16,18]. In these trials, almost all survivors were followed for 28 days and none of trials used molecular methods to differentiate between recrudescence and re-infection. In one trial the cure rate was 100% for the two arms of the trial (18). In one other trial participants received sulphadoxine/pyrimethamine (SP) before they were discharged from hospital in year two and year three of the trial [10]. In this trial, recrudescence rates in the first year were not statistically different among the two groups and in the following two years, the rates were similar (10.6% and 9.4% respectively). In the other two trials, children received SP after parasites were cleared [9,12]. In these three trials, which used SP after coma resolution, parasite clearance or at discharge, the criterion for its use was the same among trials' interventions.

**Figure 7:**
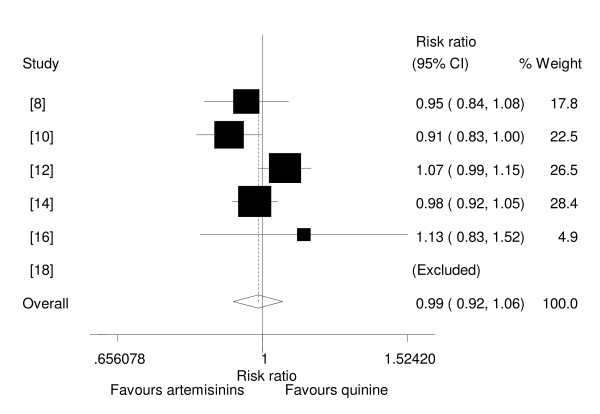
**Meta-analysis of effect of artemisinin derivatives versus quinine on 28^th ^day cure rates (random effect model). **Weight of study was expressed by size of square.

In two trials one child each, in the artemether groups, developed parasitaemia on day 14 [8,14]. These children were treated successfully. Analysis by the type of artemisinin used showed that neither β-arteether nor artemether had higher cure rate than quinine. When the results were pooled, it was shown that there was no statistical difference in cure rates between the two drug groups (R R = 0.99, 95%CI: 0.92 to 1.06, random effect model)(Figure 7). Results were similar when adequately concealed trials only were considered. Subgroup analysis to explore heterogeneity between Asian and African studies was not possible because the data available for pooling was from Africa studies only.

### Incidence of adverse effects

The trials included in this review were not designed to evaluate differences in adverse effects amongst the two groups. 11 trials commented on adverse effects. Trials, which reported this outcome, reported it either incompletely or in a way that hindered thorough comparison among the interventions. Therefore only a descriptive narrative of the data is given. One trial report contained a detailed listing of adverse effects in a trial comparing artemotil (β-arteether) with quinine as assessed twice daily for seven days and then weekly for another three weeks. It showed that there was no difference in weakness, aphasia/speech disorder, deafness, fevers/rigors, anorexia, nausea, vomiting, diarrhoea, cough, pneumonia, conjunctivitis between groups [15].

Another trial comparing artesunate with quinine found that patients on quinine suffered significantly more often from vomiting (30%), nausea (50%), headaches (40%) and tinnitus (20%) than patients on artesunate who had none of these adverse effects [18]. One trial reported on a fatal complication, which may have represented an adverse effect: A case of black water fever in a patient on quinine [16]. One trial reported that the group receiving artesunate had significantly lower reticulocytes counts compared to quinine by day 5 of treatment, but the difference was no longer significant by day of discharge [11]. Another trial reported one case of hypoglycaemia among the quinine group [17]. Four trials reported on results of ECG monitoring in 341 patients. One trial reported that patients treated with artemether showed a significant prolongation of the QTc interval significantly more frequently (20/82) than in the group treated with quinine (5/80) [9]. Another study found no significant difference in QTc intervals between groups [12] and there were more patients with prolonged QTc interval in the group on quinine. Neither of these studies demonstrated arrhythmias. Supraventricular tachycardia was noted in two patients on quinine in another study [8]. Another study reported no cardiac event in either artesunate or quinine patient after 24 hrs of cardiac monitoring from admission[11]. Another trial reported that local reactions at the site of injection were more common in the quinine group (5.9%) than in the artemether group (0.7%) with abscess formation requiring drainage in 5/288 patients in the quinine and 1/288 in the artemether group [10].

## Discussion

This review suggests that artemisinin derivatives are not superior to quinine in preventing mortality from severe malaria in childhood. It also showed that though CRT was shorter with artemisinin derivatives, this difference was no longer significant when high quality studies were considered, indicating that the overall pooled results may have been biased by results from low quality trials. Other outcomes i.e. PCT, FCT, incidence of neurological sequelae and 28^th ^cure rates showed no difference among the two drug groups. Though data on adverse effects were inadequate, administration of intramuscular quinine was characterized by more local reactions at the injection site compared to artemether. In one study, it was found that compared to quinine, administration of intramuscular artesunate led to signs of transient bone marrow depression.

Subgroup analysis of the individual artemisinins showed that none of the drug studied (i.e. β-arteether, artesunate and artemether) in this review had lower mortality rate than quinine. It is important to note that despite this conclusion there were only two studies with a total of 194 children that compared β-arteether versus quinine, and only one study with 72 children that compared artesunate versus quinine, the number of subjects in these two drugs is too small to make a conclusion on their efficacy compared to quinine in terms of mortality. In contrast there were nine studies with 1258 children that compared artemether versus quinine and found it to be not different in effect on mortality. Previous reviews also came to a similar conclusion [23,24]. A more recent review found a reduction in mortality in patients treated with artesunate [27]. A reason for the lack of mortality reduction in children treated with intramuscular artemether may be its slow and erratic absorption thus failing to reverse and prevent pathological processes leading to death in severe malaria in children. It is now known that parenteral artesunate may be faster absorbed after intramuscular injection than artemether and a study in Asia showed that it may reduce mortality in Asian adults [28]. An interest in artesunate as possible alternative to quinine led to the implementation of a big multicentre randomized study comparing injectable artesunate and quinine in children with severe falciparum malaria in Africa. When this study is completed it will be known whether artesunate is better than quinine in African children. The superiority of artemisinin derivatives in parasite clearance in trials conducted in India may be an indicator of reduced sensitivity of Asian *P. falciparum *strains to quinine. Future reviews need to include subgroup analyses for Asian and African regions separately as long as quinine resistance is sporadic in Africa.

Based on efficacy; this review does not justify a replacement of intravenous quinine by artemisinin derivatives when treating children with severe malaria in Africa and Asian countries where *P. falciparum *strains are known to be sensitive to quinine. However, since the efficacies between the two groups of drugs are comparable decision to replace quinine may be taken if other factors such as costs, user preference and logistics are taken into consideration.

Given the results of this review studies comparing artemisinin derivatives with quinine in treatment of severe malaria in children need to follow equivalence trial design. A sample size calculation for an equivalence trial taking mortality as a primary outcome, with a difference in mortality of five percent taken as range of equivalence and 85% survival as expected with standard quinine treatment of cerebral malaria, a sample size of at least 1071 in each treatment group would be required to demonstrate equivalence with a power of 80% with a two sided confidence interval of 95% for the difference in mortality [29]. For coma recovery time with a difference in coma recovery time of four hours as range of equivalence and 12 hours as standard deviation with 80% power and 95% confidence interval for the difference at least 189 participants would be required in each group to demonstrate equivalence. This means none of the trials reviewed was sufficiently powered to demonstrate equivalence and exclude clinically significant differences in these key outcomes. Adequately sized trials investigating the main outcomes mortality, coma recovery time and neurological sequelae need to be conducted in the future.

## Conclusion

There was no evidence that treatment of children with severe malaria with parenteral artemisinin derivatives was associated with lower mortality or long-term morbidity compared to parenteral quinine. Future studies require adequately powered equivalence trial design to decide whether both drugs are equally effective.

## Competing interests

The authors declare that they have no competing interests.

## Authors' contributions

GP, AD and ME contributed to the review design, GP extracted and analyzed the data. GP, AD and ME participated in interpretation of data and writing of the manuscript. All authors approved the final manuscript.

## References

[B1] WHO and UNICEF (2005). World Malaria Report 2005.

[B2] World Bank (1993). World development report 1993, investing in health.

[B3] Oaks SC, Mitchell VS, Pearson GW, Carpenter CJ (1991). Malaria: obstacles and opportunities.

[B4] Pukrittayakamee S, Supanaranond W, Looareesuwan S, Vanijanonta S, White NJ (1994). Quinine in severe falciparum malaria: evidence of declining efficacy in Thailand. Trans R Soc Trop Med Hyg.

[B5] Al-Yaman F, Genton B, Mokela D, Narara A, Raiko A, Alpers MP (1996). Resistance of *Plasmodium falciparum *malaria to amodiaquine, chloroquine and quinine in the Madang Province of Papua New Guinea, 1990–1993[abstract]. P N G Med J.

[B6] Mutanda LN (1999). Assessment of drug resistance to malaria parasite in residences of Kampala, Uganda. East Afr Med J.

[B7] Brasseur P, Kouamouo J, Brandinourst O, Moyou SR, Druilhe P (1988). Patterns of *in vitro *resistance to chloroquine, quinine and mefloquine of *Plasmodium falciparum *in Cameroon in 1985–86. Am J Trop Hyg.

[B8] Walker O, Salako LA, Omukhodion SI, Sowunmi A (1993). An open randomized comparative study of intramuscular artemether and intravenous quinine in cerebral malaria in children. Trans R Soc Trop Med Hyg.

[B9] Murphy S, English M, Waruiru C, Mwangi I, Amukoye E, Crawley J, Newton C, Winstanley P, Peshu N, Marsh K (1996). An open randomized trial of artemether versus quinine in the treatment of cerebral malaria in African children. Trans R Soc Trop Med Hyg.

[B10] Van Hensbroek MB, Onyiorah E, Jaffar S, Schneider G, Palmer A, Frenkel J, Enwere G, Forck S, Nusmeijer A, Bennett S, Greenwood B, Kwiatkowski D (1996). A trial of artemether or quinine in children with cerebral malaria. N Engl J Med.

[B11] Cao XT, Bethell DB, Pham TP, Ta TT, Tran TN, Nguyen TT, Pham TT, Nguyen TT, Day NP, White NJ (1997). Comparison of artemisinin suppositories, intramuscular artesunate and intravenous quinine for the treatment of severe childhood malaria. Trans R Soc Trop Med Hyg.

[B12] Taylor TE, Wills BA, Courval JM, Molyneux ME (1998). Intramuscular artemether vs intravenous quinine: an open, randomized trial in Malawian children with cerebral malaria. Trop Med Int Health.

[B13] Ojuawo A, Adegboye AR, Oyewalo O (1998). Clinical response and parasite clearance in childhood cerebral malaria: A comparison between intramuscular artemether and intravenous quinine. East Afr Med J.

[B14] Olumese PE, Bjorkman A, Gbadegesin RA, Adeyemo AA, Walker O (1999). Comparative efficacy of intramuscular artemether and intravenous quinine in Nigerian children with cerebral malaria. Acta Trop.

[B15] Thuma PE, Ganapati JB, Mabeza GF, Osborne C, Biemba G, Shakankale GM, Pierre AMP, Oosterhuis B, Lugt CB, Gordeuk V (2000). A randomized controlled trial of artemotil(β-arteether) in Zambian children with cerebral malaria. Am J Trop Med Hyg.

[B16] Moyou-somo R, Tietche F, Ondoa M, Kouemeni LE, Ekoe T, Mbonda E, Nsangou C, Jemea B, Guemkam G (2001). Clinical trial of β-arteether versus quinine for the treatment of cerebral malaria in children in Yaoundé, Cameroon. Am J Trop Med Hyg.

[B17] Adam I, Idris HM, Mohamed-Ali AA, A/Elbasit, Elbashir MI (2002). Comparison of intramuscular artemether and intravenous quinine in the treatment of Sudanese children with severe falciparum malaria. East Afr Med J.

[B18] Satti GM, Elhassan SH, Ibrahim SA (2002). The efficacy of artemether versus quinine in the treatment of cerebral malaria. J Egypt Soc Parasitol.

[B19] Huda SN, Shahab T, Ali SM, Afzal K, Khan HM (2003). A comparative clinical trial of artemether and quinine in children with severe malaria. Indian Pediatr.

[B20] Woodrow CJ, Haynes RK, Krishna S (2005). Artemisinins. Postgrad Med J.

[B21] Mithwani S, Aarons L, Kokwaro GO, Majid O, Muchohi S, Edwards G, Mohamed S, Marsh K, Watkins W (2004). Population pharmacokinetics of artemether and dihydroartemisinin following single intramuscular dosing of artemether in Aftrican children with severe falciparum malaria. Br J Clin Pharmacol.

[B22] Krishna S, Nagaraja NV, Planche T, Agbenyega T, Bedo-Addo G, Ansong D, Owusu-Ofori A, Shroads AL, Henderson G, Hutson A, Derendorf H, Stacpoole PW (2001). Population pharmacokinetics of intramuscular quinine in children with severe malaria. Antimicrob Agents Chemother.

[B23] Pittler MH, Ernst E (1999). Artemether for severe malaria: a meta-analysis of randomised clinical trials. Clin Infect Dis.

[B24] McIntosh HM, Olliaro P (2004). Artemisinin derivatives for treating severe malaria.

[B25] World Health Organization (2000). Severe falciparum malaria. Trans R Soc Trop Med Hyg.

[B26] Higgins JPT, Green S, editors Cochrane Handbook for Systematic Reviews of Interventions 4.2.5[Updated May 2005]. http://www.cochrane.org/resources/handbook.htm.

[B27] Jones KL, Donegan S, Lalloo DG (2007). Artesunate versus quinine for treating severe malaria. Cochrane Database Syst Rev.

[B28] South East Asian Quinine Artesunate Malaria Trial (SEAAQUAMAT) Group (2005). Artesunate versus quinine for treatment of severe falciparum malaria. a randomized trial. Lancet.

[B29] Jones B, Jarvis P, Lewis JA, Ebbutt AF (1996). Trials to assess equivalence: theimportance of rigorous methods. BMJ.

